# Integration of Carbon Dots on Nanoflower Structured ZnCdS as a Cocatalyst for Photocatalytic Degradation

**DOI:** 10.3390/ma16010366

**Published:** 2022-12-30

**Authors:** Jie Zhou, Xin Zhao, Haoming Xu, Zhichao Wang, Xiaoyuan Zhang, Zhiqiang Su

**Affiliations:** 1 State Key Laboratory of Chemical Resource Engineering, Beijing Key Laboratory of Advanced Functional Polymer Composites, Beijing University of Chemical Technology, Beijing 100029, China; 2 Precision Forestry Key Laboratory of Beijing, Beijing Forestry University, Beijing 100083, China

**Keywords:** photocatalytic degradation, carbon dots, ZnCdS, nanoflower, rhodamine B

## Abstract

The application of catalysts is one of the most effective methods in the oil refining, chemical, medical, environmental protection, and other industries. In this work, carbon dots (CDs) were selected as an initiator and doped into the main catalyst, Zn_0.2_Cd_0.8_S, and a novel Zn_0.2_Cd_0.8_S@CD composite catalyst with a nanoflower structure was successfully obtained. The synthesized composites (Zn_0.2_Cd_0.8_S@CDs) were characterized by means of SEM, TEM, XRD, FT-IR, XPS, and UV-Vis DRS. Transient photocurrent response and Nyquist curve analysis further proved that the carrier separation efficiency of the composite catalyst was significantly improved. In addition, the photocatalytic activity of Zn_0.2_Cd_0.8_S@CDs for rhodamine B removal from aqueous solution was tested under visible-light irradiation. When the amount of Zn_0.2_Cd_0.8_S@CDs composite catalyst reached 50 mg, the degradation rate of rhodamine B was 79.35%. Finally, the photocatalytic degradation mechanism of the Zn_0.2_Cd_0.8_S@CDs complex was studied. CD doping enhances the adsorption capacity of Zn_0.2_Cd_0.8_S@CDs composite catalysts due to the increase in surface area, effectively inducing charge delocalization and enhancing the photocatalytic capacity. Zn_0.2_Cd_0.8_S@CDs composites with low cost and high carrier separation efficiency have broad application prospects in the photocatalytic degradation of dyes.

## 1. Introduction

Rhodamine B (RhB) is a typical cationic organic dye that is mainly used in the fields of printing and dyeing, as well as medicine and steel. When RhB is discharged into water bodies, it turns the color of water purple, making it difficult for sunlight to reach the water, damaging the self-purification ability of the water body, and causing eutrophication [1].When the human body is exposed to RhB by mistake, it acts on the skin and blood vessels, causing symptoms such as red staining of the skin and internal organs, mild bruising of blood vessels in the brain, fracture of heart muscle fibers, and blurring and disappearance of cross lines [2]. Therefore, it is necessary to treat RhB industrial wastewater before discharge. Methods investigated for the degradation of RhB include chemical oxidation, Fenton reaction, electrochemical oxidation, and photocatalysis. Chemical oxidation [3,4] uses oxidizing agents to oxidize RhB and is prone to secondary contamination; electrochemical oxidation [5,6] is fast and simple but difficult to apply due to high energy and power consumption. Fenton reaction [7,8,9] and photocatalytic [10,11,12,13] methods use generated hydroxyl radicals to oxidize RhB, while photocatalytic method is the most studied of these methods.

Zn_x_Cd_1−x_S solid solution is a ternary photocatalyst with a controllable band gap and edge position. It has the advantages of both a narrow band gap of CdS and high stability of ZnS, in addition to good application potential in photocatalysis, especially in hydrogen production. Zeng et al. [14] doped Ag into ZnCdS semiconductor nanocrystals to improve the optical properties of the composites by mediating the Ag concentration. The addition of Ag precursors affected the relative activity of Zn and Cd precursors. With the increasing band gap of the ZnCdS nanocrystals, the excited Ag Dopant D state shifts to a lower energy level, and the dopant emission also exhibits a blue shift. The photocatalytic properties of single ZnCdS cannot meet the requirements of the present era, so it is urgent to find a composite of ZnCdS and other functional materials to improve the photocatalytic properties [15,16,17].

Carbon dots (CDs) are nano-sized new materials with excellent optical properties, good water solubility, and low toxicity, that are environmentally friendly, representing a comprehensive source of raw materials that are low-cost, biocompatible, and photoluminescent [18,19,20]. CDs have excellent electrical conductivity and charge transport ability. CDs can be used as electron donors and electron acceptors, providing a new idea for efficient light energy and catalyst design [21,22,23,24,25]. Their surface can be modified to increase the active sites, benefitting the production of free radicals or proton reduction [15,26,27,28]. Furthermore, CDs can modulate the band structure of CD-based composites and promote the absorption of light [12,20,21]. Zhu et al. [29] synthesized heteroatom-doped xylose-based carbon (X-CD) and chitosan-based carbon (C-CD) using xylose and chitosan as carbon sources and sulfoxide chloride as a dopant, respectively, to dope S elements. It should be noted that chitosan was used as both a carbon source and an endogenous nitrogen dopant. X-CD doped with only S elements was found to provide more advantages in electron-hole separation because N-related functional groups with strong electron-donating properties weaken the electron capture ability of S-related energy levels.

CDs, serving as an ideal candidate material in the field of energy and the environment, can be used as photocatalysts alone or as auxiliary catalysts for catalytic reactions with a substrate list of semiconductor materials [25,28,30,31] Huang et al. [22] doped CDs in WO_3_ nanoplates to construct the interface heterojunction from CDs to WO_3_. This electric field drives photoexcited electrons to transmit to CDs, and the remaining photoexcited holes remain on WO_3_, thus realizing the space separation of electrons and holes. Teng et al. [32] prepared CDs, MoS_2_, and p-C_3_N_5_ composites with different proportions by hydrothermal and calcination methods. The high charge mobility and high-density hydrogen evolution sites of MoS_2_ nanosheets, together with the electronic storage and transfer properties of CDs, can significantly improve the electron transport on the p-C_3_N_5_ surface and reduce the photogenerated carrier recombination. CDs/MoS_2_/p-C_3_N_5_ composites achieve good photocatalytic performance for hydrogen evolution (H_2_) and degradation of methylene blue (MB). Among them, 5% D-MoS_2_ (CMSCN5) exhibits the best photocatalytic activity. The hydrogen yield of CMSCN5 is 444 mmol/g·h, which is 1.45 times higher than that of unmodified p-C_3_N_5_, with an MB removal rate reaching 93.51% within 120 min. 

The introduction of N-doped carbon quantum dots (NCQDs) can improve the photocatalytic performance of the catalyst. Xu et al. [33] synthesized NCQDs using g-C_3_N_4_ as a carbon and nitrogen source. NCQDs with a size of 3.2 nm were prepared by hydrothermal etching and then adsorbed on the surface of CDs with a size of 80 nm to form a strong C-S bond interaction, which induced the surface morphology of CDs to change. NCQDs not only improved the photoabsorption performance of the catalyst but also made the transfer of photogenerated electrons to NCQDs rapid and effective.

In this work, Zn_0.2_Cd_0.8_S@CD catalysts were prepared by a simple one-pot hydrothermal method. Various characterization methods were applied to study the photocatalytic properties of Zn_0.2_Cd_0.8_S@CDs and the specific effects of CDs. Some defects of Zn_0.2_Cd_0.8_S are remedied by utilizing the relative advantages of CDs, such as unique fluorescence, photoluminescence, conductivity, and charge transfer ability. CDs are small in size, with highly dispersive characteristics, which do not affect the function of photocatalysis but can enhance the properties of Zn_0.2_Cd_0.8_S to optimize the photocatalytic performance. In addition, the photocatalytic degradation properties of Zn_0.2_Cd_0.8_S@CDs were evaluated for rhodamine B (RhB). The results show that the degradation of 10 mg/L RhB solution using a puppy catalyst reached 50.8% within 1 h and 71.36% within 2 h, which superior to the performance reported in other studies [34,35,36]. Finally, the photocatalytic degradation mechanism of the Zn_0.2_Cd_0.8_S@CD composite photocatalyst was explored.

## 2. Experiment

### 2.1. Materials

Citric acid, urea (99.5%), zinc acetate dihydrate (99%), thiourea (99%), sodium sulfate (99%), RhB (96%), potassium bromide (99%), and isopropyl alcohol (99.5%) were purchased from Carbaryl (Shanghai, China). N-dimethylformamide (DMF) (98%), cadmium acetate dihydrate (98%), and Nafion solution were purchased from Tianjin Damao Chemical Plant (Tianjin, China), Thermo Fisher Scientific (Beijing, China) and Tianyu Chemical Technology (Hebei, China), respectively. All of the above reagents were of analytical grade. Deionized water was produced using a Milli-Q system.

### 2.2. Synthesis of Zn_0.2_Cd_0.8_S and Zn_0.2_Cd_0.8_S@CDs

**Scheme 1:**
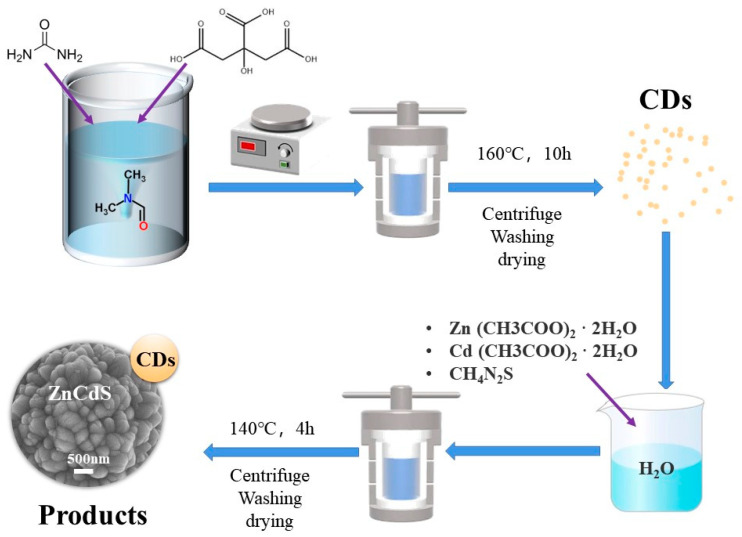
Schematic diagram of the preparation of Zn_0.2_Cd_0.8_S and Zn_0.2_Cd_0.8_S@CDs.

Zn_0.2_Cd_0.8_S was prepared by a facile one-pot hydrothermal method with Zn (CH_3_COO)_2_·2H_2_O as a zinc source, Cd (CH_3_COO)_2_·2H_2_O as a cadmium source, CH_4_N_2_S as a sulfur source, and H_2_O as a solvent. First, 0.22 g zinc acetate dihydrate, 1.07 g cadmium acetate dihydrate, and 0.38 g thiourea were weighed using an electronic analytical balance and dissolved in 80 mL of ultrapure water. The solution was homogenized by agitation and ultrasound and transferred to the polytetrafluoroethylene (PTFE) lining of a reactor. The reactor was placed in an oven at a temperature of 140 ℃ for 4 h. After the end of the reaction, the reactor was cooled, and the liquid was removed and centrifuged with a low-speed centrifuge. Then, the resulting precipitation was washed with ethanol and ultrapure water multiple times and dried in a vacuum environment at 60 ℃ for 12 h. Finally, the milled product was powdered for subsequent use. For comparison with the synthesis of Zn_0.2_Cd_0.8_S, a given amount of CDs (0, 0.13, 0.26, and 0.52 mg) prepared in advance according to reference [21] was added for the synthesis of Zn_0.2_Cd_0.8_S@CDs (Scheme 1).

### 2.3. Characterization

The samples were characterized by a Hitachi SU8010 scanning electron microscope (SEM). The microstructure was characterized by an FEI-TALOS-F200X transmission electron microscope (TEM). A UL3101-1 atomic force microscope (AFM) was used to characterize the morphology and size of CDs. An FTIR-650 infrared spectrometer and an Ultima IV X-ray diffractometer (XRD) were used to analyze the structure of the samples. The element properties were studied using a Thermo Scientific Kα X-ray photoelectron spectroscope (XPS). UV absorption spectra and UV diffuse reflectance spectra were studied with PerkinElmer and LAMADA950 UV-vis spectrometers. Steady-state, transient fluorescence spectra of samples were obtained with a Hitachi F-4600 photometer. The Mott-Schottky curve, photocurrent response and EIS Nyquist plot were measured on an electrochemical workstation (CHI760E)**.**

The photocatalytic degradation of RhB as a model dye pollutant at room temperature was studied using prepared catalysts in a homemade light reactor using a 500W xenon lamp source. In the experiment, a given amount of photocatalyst was suspended in 500 mL of 10 mg/L RhB solution. In the dark, a magnetic stirrer was used to stir the suspension at a constant speed for 30 min to achieve the adsorption–desorption equilibrium of the dye on the photocatalyst. Then, the suspension was kept in a photocatalytic reactor, the light source was turned on, magnetic stirring was continued, and 5 mL suspension was removed every 30 min and centrifuged at 3000 rpm. The resulting solution was analyzed by a UV-visible spectrophotometer (UV-3150, Shimadzu, Tokyo, Japan) after centrifugation. The concentration of RhB dye was quantified by measuring the absorbance at 553 nm.

The following formula is used to calculate the degradation rate:

(1) $$D\left(\%\right)=\left(C_{0}-C\right)/C_{0}$$

where D represents the degradation rate (%), *C*_0_ is the initial concentration of RhB (mg/L), and *C* is the immediate concentration of RhB every 30 min (mg/L).

## 3. Results and Discussion

### 3.1. Morphologic Analysis of the Zn_0.2_Cd_0.8_S@CD Catalyst 

**Figure 1:**
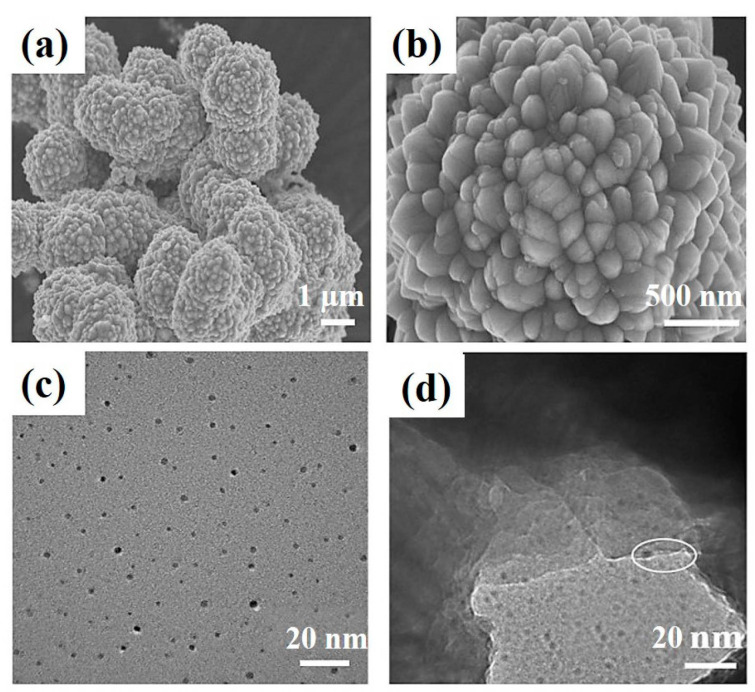
(**a**,**b**) SEM image of Zn_0.2_Cd_0.8_S; (**c**) TEM image of CDs; (**d**) TEM image of Zn_0.2_Cd_0.8_S@CDs.

The morphology of Zn_0.2_Cd_0.8_S was observed by SEM. As shown in Figure 1a, the morphology of Zn_0.2_Cd_0.8_S shows a nanoflower structure with obvious agglomeration, and the size is approximately 4 μm. Figure 1b shows an enlarged view of a single Zn_0.2_Cd_0.8_S, which indicates the microstructure. The microstructure of Zn_0.2_Cd_0.8_S shows a large specific surface area, which is advantageous for catalytic processes, as it often contains more active catalytic sites [37]. Zn_0.2_Cd_0.8_S exhibited a combination of type I and type IV isotherms based on the IUPAC classification [38], indicating a predominance of micropores and mesopores, with an SBET of 4.8945 m^2^/g (Figure S1c), possibly because the SBET of nanoflowers composed of columnar granular “petals” is lower than that of nanoflowers composed of flaky “petals”. Nevertheless, Zn_0.2_Cd_0.8_S@CDs still exhibited high catalytic activity. Owing to the small size of CDs, their microstructure was characterized by TEM. It is evident from Figure 1c that the CDs are evenly spaced out and have diameters between 3 and 4 nm. Figure 1d is a TEM map of Zn_0.2_Cd_0.8_S@CDs, showing that CD nanoparticles successfully attach to Zn_0.2_Cd_0.8_S. The close interaction between Zn_0.2_Cd_0.8_S@CDs reveals the fast transfer of interface charge and a reduction in the recombination of photogenerated carriers. However, the Zn_0.2_Cd_0.8_S catalyst may also be too thick for electrons to penetrate, resulting in black areas in the TEM map. Figure S1a shows that CDs are uniformly dispersed, which is consistent with the TEM results. The size of the synthetic CDs increased slightly due to agglomeration (Figure S1b).

### 3.2. Structural Analysis of the Zn_0.2_Cd_0.8_S@CD Catalyst 

**Figure 2:**
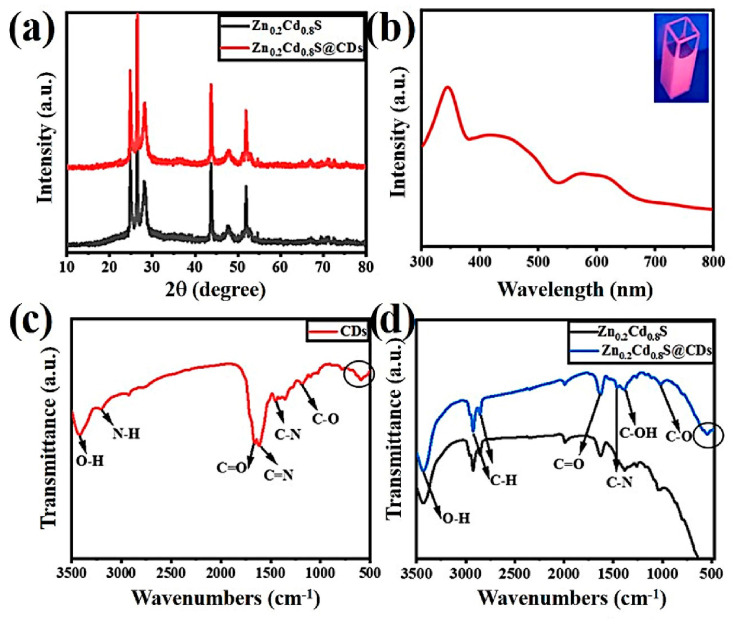
(**a**) XRD patterns of Zn_0.2_Cd_0.8_S and Zn_0.2_Cd_0.8_S@CDs; (**b**) UV-vis spectra of CDs; (**c**,**d**) FTIR spectrum of Zn_0.2_Cd_0.8_S CDs and Zn_0.2_Cd_0.8_S@CD composite.

The XRD patterns of the prepared Zn_0.2_Cd_0.8_S and the resulting Zn_0.2_Cd_0.8_S@CDs are depicted in Figure 2. The diffraction peaks of Zn_0.2_Cd_0.8_S are strong and narrow, which shows very strong crystallinity. The diffraction peaks at 25.19°, 26.62°, 28.16°, 43.56°, 47.99°, and 51.95° correspond to the crystal faces of Zn_0.2_Cd_0.8_S (100), (002), (101), (110), (103), and (200). The addition of CDs has little effect on the lattice of Zn_0.2_Cd_0.8_S (Figure 2a) because the surface coupling of CDs has no significant effect on the intrinsic optical properties of Zn_0.2_Cd_0.8_S. CDs are most likely present on the surface of Zn_0.2_Cd_0.8_S nanoparticles rather than doped into the Zn_0.2_Cd_0.8_S lattice. In addition, the average particle size of the obtained nanoparticles was preliminarily calculated using the Debye–Scherrer formula based on the X-ray diffraction results. In the formula, D = kλ/βCosθ, D is the mean particle size (nm), λ is the wavelength of the X-ray source (1.5406 A for Cu kα), k is Debye–Scherrer constant (0.94), β is the full width at half maximum (FWHM) of the diffraction peaks, and θ is half of the diffraction angle. The calculated diameter of Zn0.2Cd0.8S and Zn0.2Cd0.8S@CDs nanoparticles is estimated to be 25.7 and 33.9 nm, respectively [24,25]. The UV-vis absorption spectrum of CDs in Figure 2b shows that the maximum absorption wavelength of CDs is 365 nm under UV irradiation, and at this wavelength, the CD solution exhibits a strong red fluorescence (as shown in the illustration).

The measured FTIR spectra of CDs, Zn_0.2_Cd_0.8_S, and Zn_0.2_Cd_0.8_S@CDs are shown in Figure 2c,d, respectively. Specifically, tensile vibration peaks of O–H and N–H can be observed in Figure 2c at approximately 3400 cm^−1^ and 3200 cm^−1^, respectively, tensile vibration peaks of C–H at approximately 2900 cm^−1^. The characteristic peak of the C=O bond appears at 1666 cm^−1^. The typical peak of the C=N bond appears at 1446 cm^−1^, and the typical peak of the C–N bond appears at 1612 cm^−1^ due to the synthesis of CDs using DMF as a solvent. The characteristic peak of C–O occurs at 1186 cm^−1^, indicating that there are many hydroxyl groups in the structure of fluorescent CDs. Figure 2d shows that the infrared spectrum of the composite Zn_0.2_Cd_0.8_S@CDs is approximately the same as that of Zn_0.2_Cd_0.8_S. The O–H stretching vibration peak is at approximately 3440 cm^−1^. The peaks at 2950 cm^−1^ and 2856 cm^−1^ represent the stretching vibration of C–H. The tensile vibration peak of C=O occurs at 1623 cm^−1^, and the stretching vibration peak of C–OH occurs at 1378 cm^−1^, both of which are associated with cadmium acetate and zinc acetate. However, the peak of Zn_0.2_Cd_0.8_S@CDs at 1450 cm^−1^, which is a C–N stretching vibration peak, is higher than the peak of Zn_0.2_Cd_0.8_S. Furthermore, the peak of CDs at 560 cm^−1^ also appears in the infrared spectrum of Zn_0.2_Cd_0.8_S@CDs, which means the CDs were successfully complexed with Zn_0.2_Cd_0.8_S.

**Figure 3:**
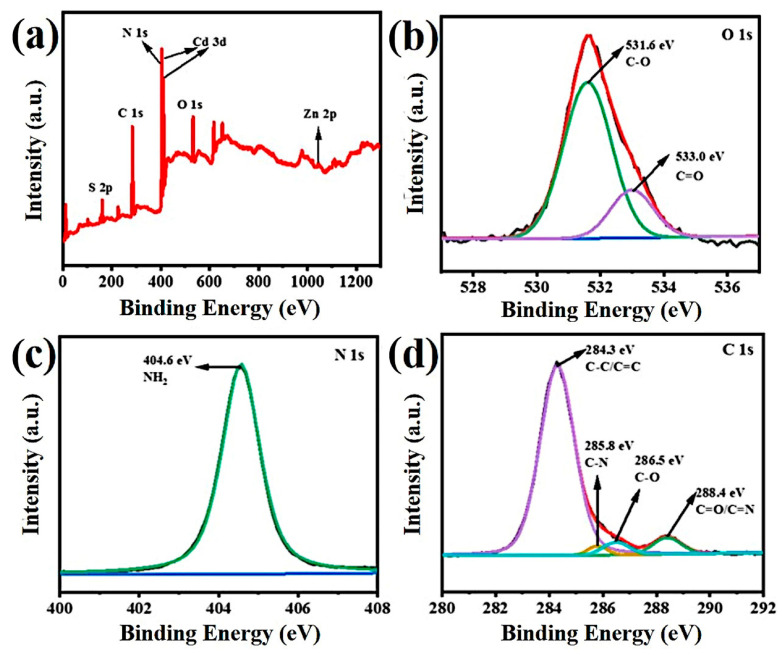
(**a**) Full scan XPS spectrum of the Zn_0.2_Cd_0.8_S@CD composite; high-resolution XPS O 1s; (**b**) N 1s; (**c**) C 1s; (**d**) spectrum of Zn_0.2_Cd_0.8_S@CDs.

Figure 3a shows the XPS full spectrum of the Zn_0.2_Cd_0.8_S@CD complex, showing the presence of Zn, Cd, S, C, N, and O elements in the composite, which is very close to the XPS full spectrum of Zn_0.2_Cd_0.8_S (Figure S3a). In the high-resolution O 1s spectrum of Zn_0.2_Cd_0.8_S@CDs (Figure 3b), the peaks at 531.6 and 533.0 eV are attributed to the C–O and CO bonds, respectively, with some negative shifts compared to Figure S2c, and this shift may be caused by the strong electron interaction between Zn_0.2_Cd_0.8_S and CDs. In the high-resolution N 1s spectrum (Figure 3c), the peak located at 404.6 eV corresponds to the amino peak. The N 1s binding energy of Zn_0.2_Cd_0.8_S@CDs is greatly increased compared to the N 1s spectrum of CDs (Figure S2d), indicating some coupling between Zn_0.2_Cd_0.8_S and CDs. This contributes to the effective interfacial charge separation and transfer between Zn_0.2_Cd_0.8_S and CDs. Figure 3d shows that C1s can be divided into four peaks: 284.3 eV, 285.8 eV, 286.5 eV, and 288.4 eV, corresponding to a C–C/C=C bond, C–N bond, C–O bond, and C=O/C=N bond, respectively. The corresponding peaks at the C–N, C–O, and C=O/C=N bonds are weakened compared to the C1s spectrum in Figure S2b, which indicates that some carbon-containing functional groups were removed during the hydrothermal process. The binding energies of Cd 3d3/2 (Figure S3c) and S 2p1/2 (Figure S3d) in the complexes are slightly shifted by 0.1 eV compared to pure Zn_0.2_Cd_0.8_S, with enhanced binding energy, indicating that electrons are lost and transferred to CDs.

**Figure 4:**
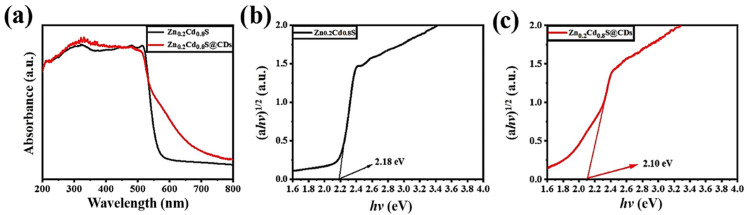
(**a**) UV-Vis DRS absorption spectra of Zn_0.2_Cd_0.8_S and Zn_0.2_Cd_0.8_S@CDs; Band gap energy of (**b**) Zn_0.2_Cd_0.8_S and (**c**) Zn_0.2_Cd_0.8_S@ CDs.

UV-Vis DRS spectroscopy was used to study the light absorption capacity of photocatalysts. Figure 4a shows the light absorption capacity information of Zn_0.2_Cd_0.8_S and Zn_0.2_Cd_0.8_S@CDs. Zn_0.2_Cd_0.8_S and Zn_0.2_Cd_0.8_S@CDs have strong light-trapping ability in the 200–550 nm wavelength range. To some extent, the light-trapping ability of Zn_0.2_Cd_0.8_S@CDs is better than that of Zn_0.2_Cd_0.8_S. According to the Formulas (2) and (3) shown below, the band gaps of three catalysts were obtained from the Tauc curve [26,27]:

(2) $${\left(\alpha hv\right)}^{1/2}=\;K\;\left(hv-E_{g}\right)$$

(3) hv=1240/λ

where *α* is absorption coefficient, *h* is the Planck constant, *v* is the frequency, K is the constant, *E_g_* is the semiconductor bandgap width, and λ is wavelength (nm). 

The band gaps of Zn_0.2_Cd_0.8_S and Zn_0.2_Cd_0.8_S@CDs are 2.18 eV (Figure 4b) and 2.10 eV (Figure 4c), respectively, indicating that CDs are more easily activated from the conduction band to the valence band and enhance the efficient utilization of light by Zn_0.2_Cd_0.8_S.

**Figure 5:**
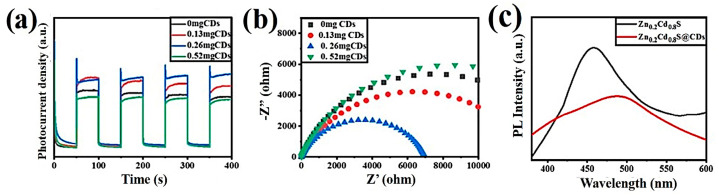
(**a**) Instantaneous photocurrent diagram of Zn0.2Cd0.8S@CDs with changes in the quantity of CDs quantity; (**b**) electrochemical impedance diagram of Zn0.2Cd0.8S@CDs with changes in the quantity of CDs; (**c**) steady-state fluorescence spectra of Zn0.2Cd0.8S and Zn0.2Cd0.8S@CDs.

The recombination of electron holes and the electron transfer rate in photocatalytic materials were investigated by transient photocurrent and electrochemical impedance measurements (Figure S4). The effect of different contents of CDs on the photocatalytic properties of Zn_0.2_Cd_0.8_S@CDs can be obtained from Figure 5a,b. Figure 5a shows that the intensity of instantaneous photocurrent increases first and then decreases with an increase in CD content. Low resistance makes the electrons transfer faster when they are excited, resulting in low electron-hole recombination efficiency. As shown in Figure 5b, the arc radius of the Nyquist graph decreases first and then increases with an increase in the amount of added CDs, which means that the changing trend of electrode resistance decreases first and then increases. The smallest semicircle in the figure corresponds to 0.26 mg of added CDs is, indicating that Zn_0.2_Cd_0.8_S@CDs have the lowest electron transfer resistance and the highest electron transfer rate.

Information about separation and transfer of charge and electron-hole recombination in a photocatalyst can be obtained by PL spectra. The PL intensity is positively correlated with the electron-hole recombination rate; the lower the catalyst’ s fluorescence intensity, the higher the photocatalytic activity [39]. As shown in Figure 5c, Zn_0.2_Cd_0.8_S has a significant PL peak at 457 nm. This higher strength indicates that the electron-hole recombination Zn_0.2_Cd_0.8_S of is more obvious. However, the fluorescence intensity of the composite catalyst with CDs is lower, which means that the photoexcited electrons transfer from the catalyst to the CDs, indicating that there is less recombination of electrons and holes in Zn_0.2_Cd_0.8_S@CDs. The coupling between CDs and Zn_0.2_Cd_0.8_S provides a fast electron transfer pathway, which improves the photocatalytic properties of Zn_0.2_Cd_0.8_S@CDs composites.

### 3.3. Study of Photocatalytic Degradation of RhB

The absorbance of the solution containing RhB was measured at a wavelength of 553 nm using a UV-Vis spectrophotometer (Figure S5a). A graph of the absorbance at a wavelength of 553 nm versus RhB concentration is shown in Figure S5b. It is obvious that the curve fits the standard curve of the first-order function, and the linear fitting Equation (4) was obtained for subsequent calculation of the degradation rate and instant concentration of RhB:

(4) Y=0.20353X+0.077

where X is the instant concentration of RhB, and Y is the absorbance of RhB at a wavelength of 553 nm. 

**Figure 6:**
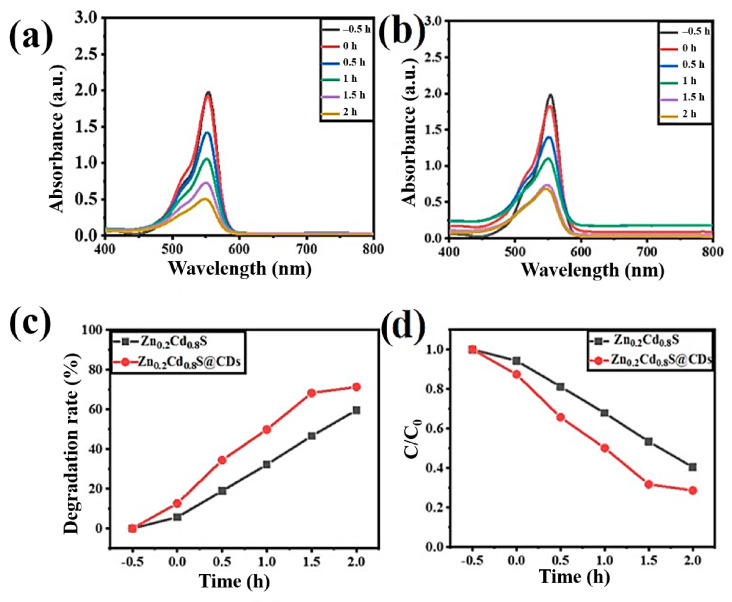
The intensity of the absorption peaks of RhB dye in the presence of Zn_0.2_Cd_0.8_S@CDs (**a**) and Zn_0.2_Cd_0.8_S (**b**) varies with time. (**c**) The degradation rate of RhB varies with time under the action of catalysts. (**d**) The concentration rate of RhB varies with time under the action of catalysts.

The absorbance of the RhB solution containing 30 mg of Zn_0.2_Cd_0.8_S@CDs (Figure 6a) and Zn_0.2_Cd_0.8_S (Figure 6b) decreased every half hour as a result of the degradation of RhB. In Figure 6c, the Zn_0.2_Cd_0.8_S@CD composite catalyst shows strong adsorption performance under the condition of a dark adsorption equilibrium. The degradation rate of RhB was 12.60% in the RhB-Zn_0.2_Cd_0.8_S@CD solution but only 5.70% in RhB-Zn_0.2_Cd_0.8_S solution. In addition, it should be emphasized that the degradation rate of RhB in RhB-Zn_0.2_Cd_0.8_S@CD solution was higher within 0–1.5 h than that after 1.5 h–2 h, which indicates that the effect of photocatalysis was maximized. The final degradation rate of RhB was 71.3% in RhB-Zn_0.2_Cd_0.8_S@CD solution but reached only 59.60% in RhB-Zn_0.2_Cd_0.8_S solution after two-hour irradiation. As shown in Figure 6d, under the condition of dark equilibrium adsorption, adding Zn_0.2_Cd_0.8_S@CD catalyst resulted in a more significant decrease in the concentration of RhB than adding Zn_0.2_Cd_0.8_S. The result of the photocatalytic experiments show that the photocatalytic efficiency of the composite catalyst is always higher than that of the single catalyst, which was negatively correlated with the concentration change of RhB. The final concentration of RhB was 4.04 mg/L in RhB-Zn_0.2_Cd_0.8_S solution and 2.86 mg/L in RhB-Zn_0.2_Cd_0.8_S@CD solution. It can be concluded that the combination of Zn_0.2_Cd_0.8_S and CDs can improve the separation of photogenerated electron-hole pairs, as well as the ability to collect visible light and effective photodegradation of RhB.

**Figure 7:**
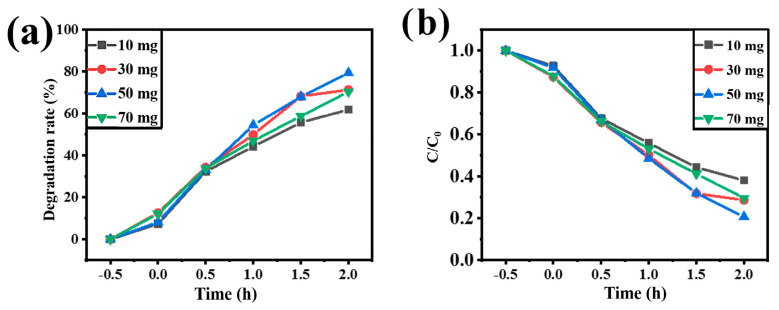
(**a**) Curves of the degradation rate of RhB with different dosages of Zn_0.2_Cd_0.8_S@CD photocatalyst over time; (**b**) concentration curves of RhB with different dosages of Zn_0.2_Cd_0.8_S@CDs photocatalyst over time.

We explored the effects of different dosages of Zn_0.2_Cd_0.8_S@CDs (10 mg, 30 mg, 50 mg, and 70 mg) on the photocatalytic degradation ability of RhB. As shown in Figure 7a, the degradation rate of RhB first increased and then decreased with increased content of Zn_0.2_Cd_0.8_S@CDs after two-hour irradiation. The highest degradation of RhB was caused by 50 mg of Zn_0.2_Cd_0.8_S@CDs. Furthermore, as shown in Figure 7b, when the dosage of Zn_0.2_Cd_0.8_S@CDs was 50 mg, the concentration of RhB solution was the lowest after irradiation for 2 h. These results indicate that 50 mg of Zn_0.2_Cd_0.8_S@CDs had the most obvious photocatalytic degradation effect on RhB. The increased dosages of Zn_0.2_Cd_0.8_S@CDs provided more active sites and thus improved the efficiency of photocatalytic degradation of RhB. However, with continued addition of catalyst, the Zn_0.2_Cd_0.8_S@CDs particles began to aggregate, resulting in the active sites becoming covered and inactivated in the solution. The above results indicate that the selection of appropriate catalyst content can effectively improve the photocatalytic degradation performance of Zn_0.2_Cd_0.8_S@CDs.

### 3.4. Mechanism Exploration 

**Figure 8:**
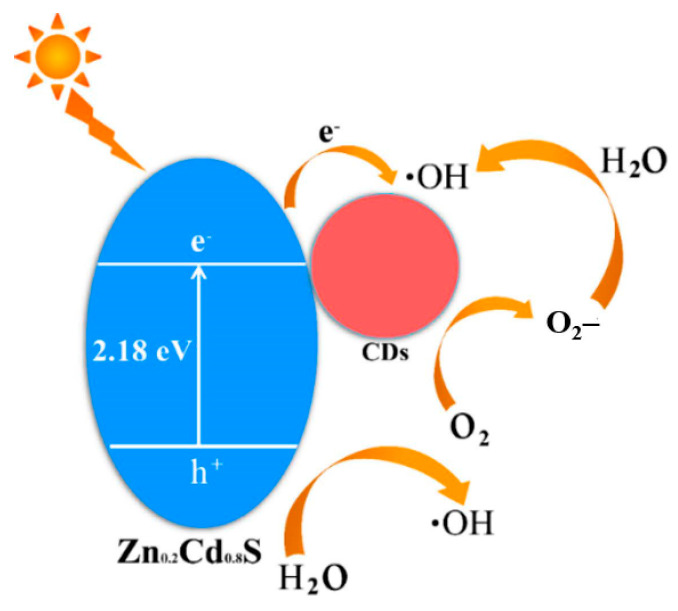
Schematic diagram of charge separation and transfer in Zn_0.2_Cd_0.8_S@CDs.

Figure 8 shows the photocatalytic degradation mechanism of RhB under simulated direct sunlight. Valence electrons of Zn_0.2_Cd_0.8_S are excited when Zn_0.2_Cd_0.8_S is irradiated by sunlight. They move towards the conduction band of Zn_0.2_Cd_0.8_S, forming electron-hole pairs [28,29]. In general, photoinduced electrons and holes tend to recombine as quickly as possible, and only a limited number of electrons and holes is available for photocatalytic reactions. The doping of CDs accelerates the transfer of electrons and reduces the recombination of electron holes. Then, electrons transfer from the Zn_0.2_Cd_0.8_S conduction band to CDs and react with adsorbed O_2_ to obtain a superoxide radical (•O^2−^). The valence band holes in Zn_0.2_Cd_0.8_S react with the adsorbed H_2_O molecules to generate hydroxyl radicals (•OH). These hyperactive free radicals are the main reason for the effective degradation of RhB [30]. CDs plays an important role in the entire photocatalytic system; with excellent upconversion performance, they can absorb long-wavelength light and convert it into short-wavelength light, continuously provide efficient absorption of Zn_0.2_Cd_0.8_S, and greatly improve the utilization rate of solar energy [31]. The raw materials used to prepare CDs are citric acid and urea, which are abundant in N. More N-doped CDs can effectively induce charge delocalization and enhance photocatalytic activity [32]. In conclusion, a CD-based composite catalyst enhances the degradation of RhB to a certain extent, leading to a decrease in electron-hole recombination probability and the acceleration of charge transfer [33].

## 4. Conclusions

In summary, to improve the defects of Zn_0.2_Cd_0.8_S, such as fast recombination of electron-hole pairs and low absorption of visible light, a Zn_0.2_Cd_0.8_S@CDs composite catalyst was synthesized by a simple one-pot hydrothermal method with CDs as additives. The doping of CDs inhibits the recombination of electron holes, enhances the electron transfer rate, and enhances the absorption efficiency of light. The most appropriate content of CDs in the Zn_0.2_Cd_0.8_S@CD composite catalyst was 0.26 mg. The photocatalytic activity of the Zn_0.2_Cd_0.8_S@CD composite catalyst was evaluated by photocatalytic degradation of RhB. The degradation rate of RhB with 30 mg of Zn_0.2_Cd_0.8_S@CD catalyst reached 71.36%, whereas that with 30 mg of Zn_0.2_Cd_0.8_S catalyst was only 59.60%. The degradation rate of RhB with 50 mg of Zn_0.2_Cd_0.8_S@CDs reached 79.35%, which was better than that with other photocatalysts [34,35,36]. We hope that this work will lead to promising designs of novel co-photocatalysts, as well as applications in water purification by using solar visible-light photocatalysis.

## Supplementary Materials

The following supporting information can be downloaded at: https://www.mdpi.com/article/10.3390/ma16010366/s1, Figure S1: AFM image and N_2_ adsorption–desorption isotherms; Figure S2: High-resolution XPS spectrum of carbon dots; Figure S3: High-resolution XPS spectrum of ZnCdS; Figure S4: Instantaneous photocurrent diagram; Figure S5: Absorbance curve of rhodamine B.
