# Supplementary material for: Integration of Carbon Dots on Nanoflower Structured ZnCdS as a Cocatalyst for Photocatalytic Degradation
Source: Materials (Basel). 2022 Dec 30;16(1):366. doi: 10.3390/ma16010366 (PMC9821953; doi:10.3390/ma16010366)
Supplement: Supplementary file 1 [file materials-16-00366-s001.zip › materials-2082104-supplementary.pdf]

Supporting Information

Integration of Carbon Dots on Nanoflower Structured ZnCdS as A Cocatalyst for Photocatalytic Degradation

Jie Zhou[1], Xin Zhao[1], Haoming Xu[1], Zhichao Wang[2], Xiaoyuan Zhang*[,1], Zhiqiang Su*[,1]

1. State Key Laboratory of Chemical Resource Engineering, Beijing Key Laboratory of Advanced Functional Polymer Composites, Beijing University of Chemical Technology, Beijing 100029, China.

2. Precision Forestry Key Laboratory of Beijing, Beijing Forestry University, Beijing 100083, China.

*Corresponding authors. E-mail addresses: 2022500116@buct.edu.cn (X.Z.) suzq@mail.buct.edu.cn (Z.S.)

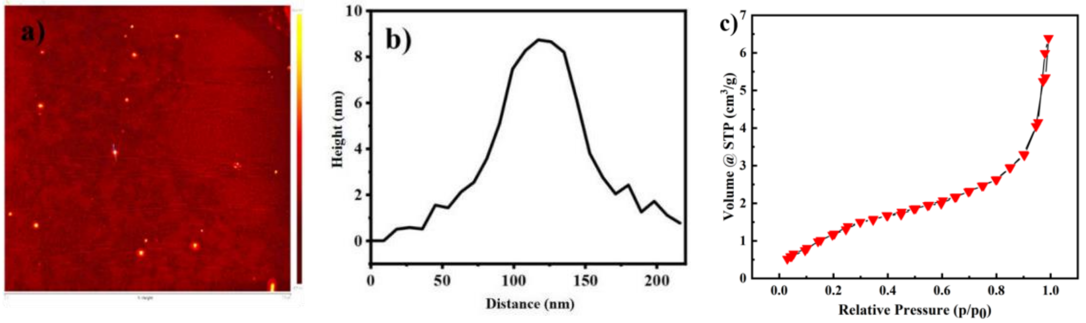

Figure S1. (a) AFM image of carbon-dots (CDs); (b) Particle size height map of carbon dots (CDs); (c) N_2 adsorption–desorption isotherms for the $Zn_{0.2}Cd_{0.8}S$ samples.

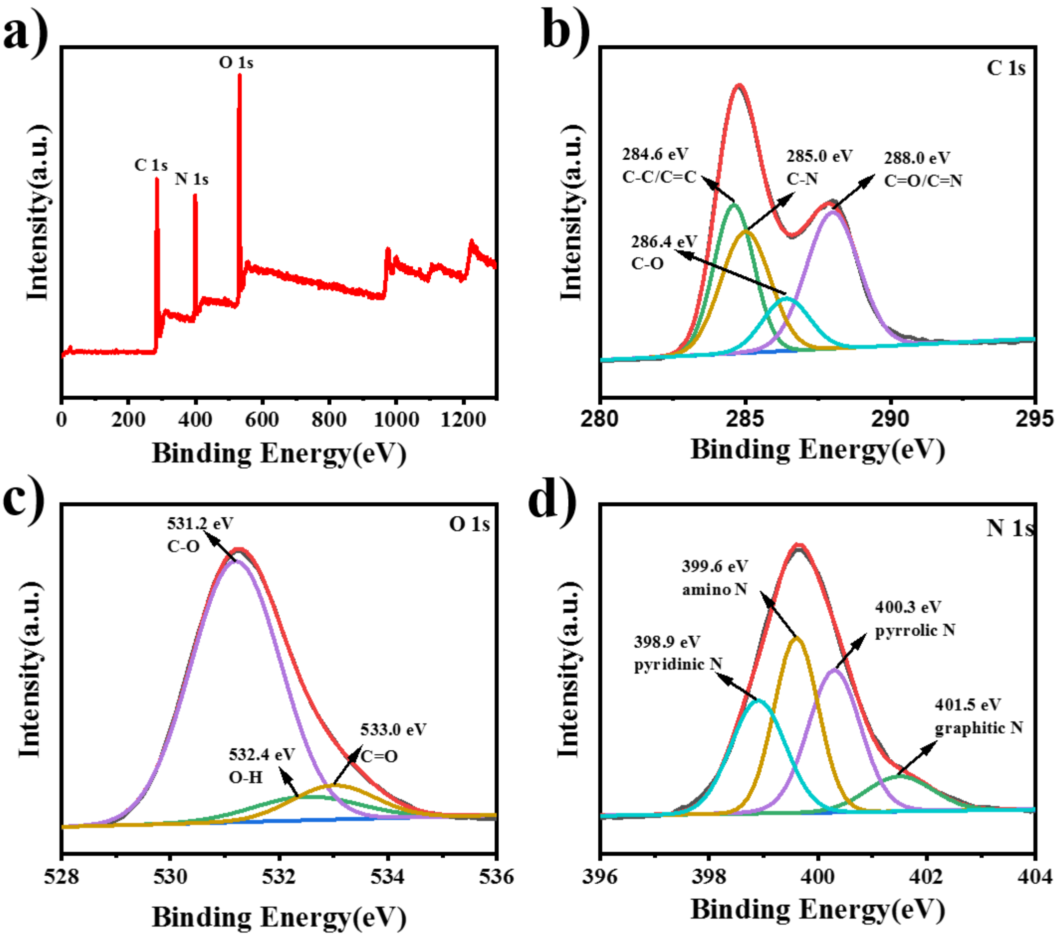

Figure S2. (a) Full scan XPS spectrum carbon dots (CDs); High-resolution XPS (b) C 1s; (c) O 1s; (d) N 1s spectrum of Carbon dots (CDs)

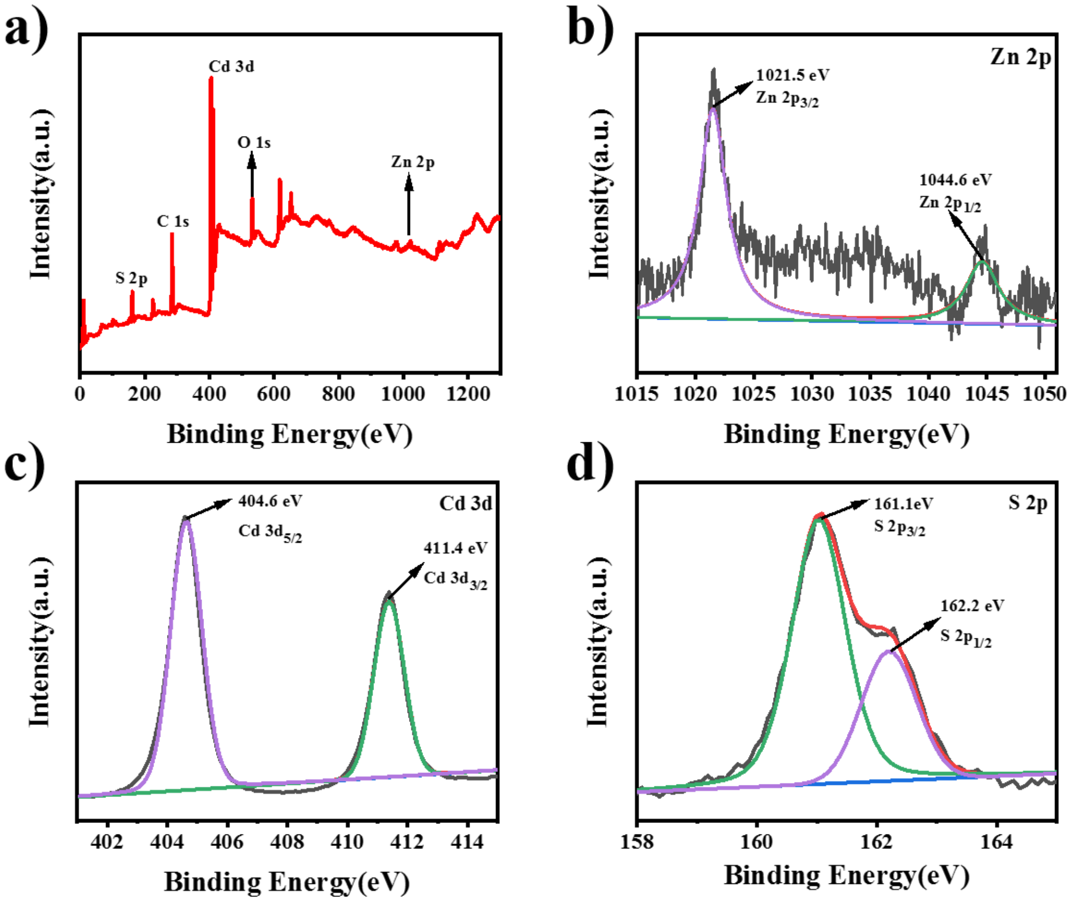

Figure S3. (a) Full scan XPS spectrum of $Zn_{0.2}Cd_{0.8}S$; High-resolution XPS spectrum of $Zn_{0.2}Cd_{0.8}S$: (b) Zn 2p; (c)Cd 3d; (d) S 2p.

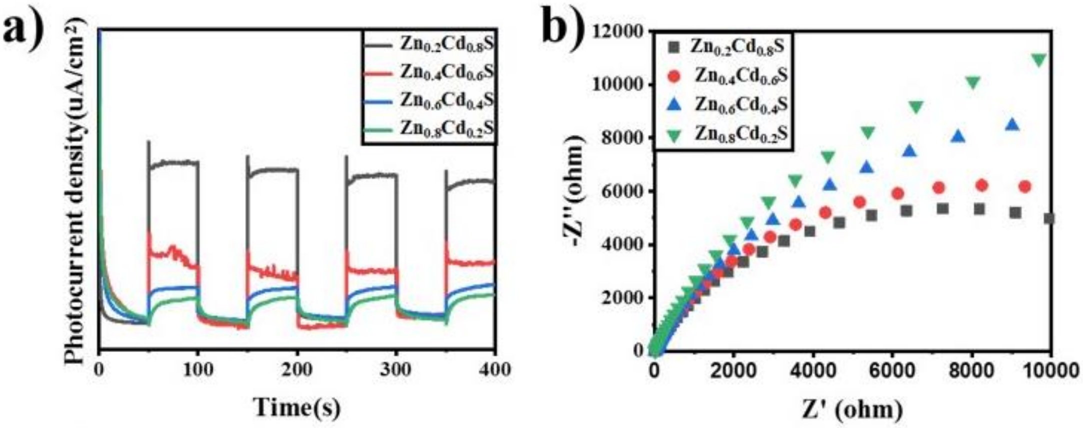

Figure S4. (a) The instantaneous photocurrent diagram of $Zn_xCd_{1-x}S$ (x=0.2, 0.4, 0.6, 0.8); (b) Electrochemical impedance plots of $Zn_xCd_{1-x}S$ (x=0.2, 0.4, 0.6, 0.8);

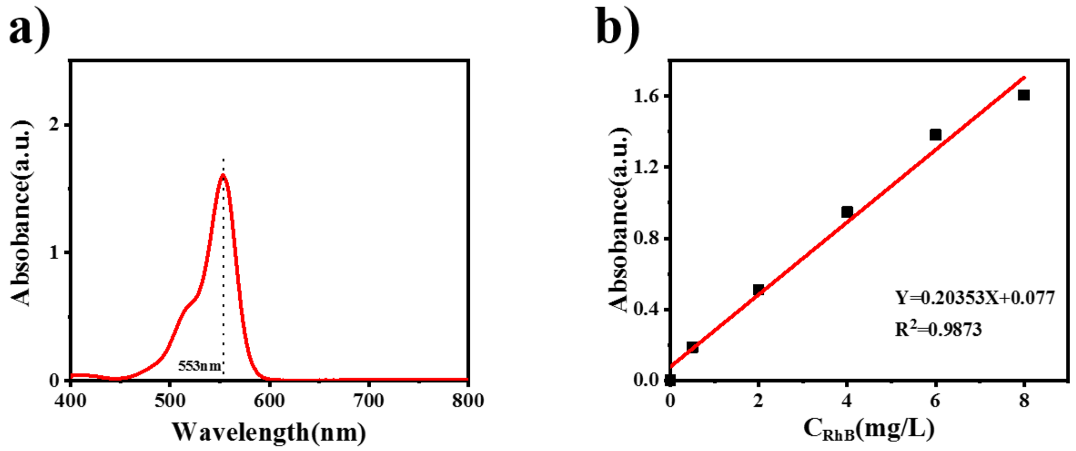

Figure S5. (a) Absorbance curve of rhodamine B solution; (b) Standard absorption curve of RhB solution
